# Maternal and Fetal Outcomes of Preeclampsia With and Without Severe Features in King Abdulaziz University Hospital, Jeddah, Saudi Arabia: A Retrospective Study

**DOI:** 10.7759/cureus.31013

**Published:** 2022-11-02

**Authors:** Aya Mousa, Rahaf L Mandili, Maysam Aljahdali, Shahlaa Gari, Sara Khaimi, Salma Alahdal, Rafeef M Derar, Sara Marzook

**Affiliations:** 1 General Practice, King Abdulaziz University Faculty of Medicine, Jeddah, SAU; 2 Medical Student, King Abdulaziz University Faculty of Medicine, Jeddah, SAU; 3 Urogynecology, King Abdulaziz University Hospital, Jeddah, SAU

**Keywords:** antenatal follow-up, preeclampsia outcomes, developing countries, preeclampsia without severe features, preeclampsia with severe features, saudi arabia

## Abstract

Background

Preeclampsia is a substantial pregnancy burden that may lead to poor feto-maternal outcomes, particularly in developing countries. It complicates 8% of pregnancies worldwide, resulting in high feto-maternal morbidity and mortality if not appropriately addressed. Despite significant development in obstetric care management in the Kingdom of Saudi Arabia (KSA), prompt recognition of the problem and the necessary care and expertise are vital to tackle this complication. Hence, we studied feto-maternal outcomes in our setting. This study aims to assess the maternal and fetal outcomes of preeclampsia with and without severe features in King Abdulaziz University Hospital (KAUH), Jeddah, KSA. This study aims to identify the maternal and fetal outcomes of preeclampsia among pregnant patients in KAUH, evaluate the role of antepartum follow-up on maternal and fetal outcomes of preeclampsia, and correlate the maternal and fetal complications of preeclampsia.

Methodology

A retrospective, cross-sectional, hospital-based study was conducted in KAUH, Jeddah, KSA. A total of 83 patients (mean age: 32 ± 6.28) who were admitted with the diagnosis of preeclampsia from 2019 to 2021 were included in the study. Feto-maternal-related outcomes were analyzed in terms of complications. A descriptive statistical method was utilized for the analysis.

Results

The mean gestational ages where preeclampsia developed with and without severe features were 33.32 weeks and 36 weeks, respectively. There were no significant relationships between preeclampsia and neonatal outcomes. However, there was a significant relationship between preeclampsia with severe features and overall maternal complications without an inclination to a specific complication. Overall, 62% of patients who did not undergo an antenatal follow-up developed preeclampsia with severe features, and 72.2% of these cases were admitted through the emergency department.

Conclusions

Preeclampsia can have life-threatening effects on both the mother and the fetus; thus, follow-ups and early recognition are recommended to drastically improve morbidity and mortality and provide effective management in line with international and national guidelines to reduce the likelihood of unfavorable outcomes.

## Introduction

Preeclampsia is a multisystem, hypertensive, pregnancy-induced disorder that causes morbidity and mortality, especially in developing countries [1-3]. It affects both mothers and their neonates [4]. Chronic hypertension is defined as high blood pressure (≥140 mmHg systole and ≥90 mmHg diastole) measured in the clinic during two or three different visits at intervals of one to four weeks [5]. High blood pressure that meets the previous criteria before 20 weeks of gestation or persists after 12 weeks postpartum indicates the diagnosis of chronic hypertension [6]. Gestational hypertension is defined as elevated blood pressure (≥140/≥90 mmHg) after 20 weeks of gestation without evidence of proteinuria and end-organ damage. When the blood pressure normalized within three months postpartum the diagnosis changes to transient hypertension of pregnancy [7].

The diagnosis of preeclampsia is established when high blood pressure (≥160/≥110 mmHg) is evident in two readings after 20 weeks of gestation or high blood pressure (≥140/≥90 mmHg) on two or more occasions at an interval of four hours. Other diagnostic factors include proteinuria or the presence of one of the following: thrombocytopenia, renal impairment, liver impairment, pulmonary edema, or persistent neurological symptoms [6]. According to contemporary research, proteinuria is no longer an essential feature to diagnose preeclampsia [8]. The diagnosis of preeclampsia is categorized into two categories based on the associated features, namely, preeclampsia without severe features and preeclampsia with severe features. The last is preeclampsia with one of the following: severely high blood pressure (≥160/≥110 mmHg), thrombocytopenia (platelet count <100,000/µL), pulmonary edema, neurological symptoms (headache or visual disturbance), elevation in serum transaminase level double the upper limit of normal, persistent right upper quadrant pain not explained by other diagnoses, oliguria (less than 500 mL per day), or significant elevation in serum creatinine not attributed to other causes [6].

Hypertensive disorders during pregnancy can be categorized based on the onset, whether it began before pregnancy, in the first 20 weeks of gestation, or the weeks after [9]. Thus, regular antenatal follow-up visits are necessary to determine the etiology and severity of hypertension during pregnancy and avoid the risk of misdiagnosing the different hypertensive disorders in pregnancy [10,11].

According to the National Institute for Health and Care Excellence (NICE) guidelines, preeclampsia risk factors can be classified as high and moderate risk factors [12]. Some of them are related to maternal medical conditions such as chronic (preexisting) hypertension, diabetes mellites, autoimmune diseases, and chronic kidney diseases. Other factors are related to the obstetric history such as a history of hypertensive pregnancy disorder, nulliparous older than 40 years old, a 10-year interval since the last pregnancy, and multiple pregnancies. In addition, some infections have been linked to preeclampsia such as urinary tract infections [13,14].

Preeclampsia complicates 8% of pregnancies globally and causes almost 15% of maternal mortality [15]. Incidence also varies ethnically with a higher rate in black women compared to white women [16,17]. In Sub-Saharan Africa, preeclampsia and eclampsia cases were included among the 80% leading causes of maternal mortality [18]. Approximately four out of 100 pregnancies in the United States are associated with preeclampsia [19]. Meanwhile, in Saudi Arabia, preeclampsia impacts approximately 5.37 per 10,000 women [20].

The fetal and maternal health can be sometimes at odds due to the various complications of preeclampsia, namely, hypertension, proteinuria, renal impairment, liver dysfunction, HELLP syndrome (hemolysis, elevated liver enzymes, low platelet count) which is a variant of preeclampsia when accompanied with hypertension, headaches, visual disturbances, and eclampsia (preeclampsia associated with unexplained generalized seizures) [1,6]. A study published in 2013 revealed that out of a total of 430 women, 14 showed advancing preeclampsia, 4.9% showed liver impairment, 12.3% showed kidney impairment, 7% showed detachment of the placenta, and 0.7% cases had an epileptic episode. There were no cases of preeclampsia-associated blindness. Additionally, a commonly occurring complication of preeclampsia, known as HELLP syndrome, was evident in 4.2% of the cases, and pulmonary edema cases in 5.6% of cases. Moreover, disseminated intravascular coagulation (DIC) was associated with 2.8% of the cases, and one case presented with encephalopathy. Cases with mild preeclampsia represented 86.5% of the total cases [21]. Fetal risks can include progressing from preeclampsia to eclampsia, HELLP syndrome, abruptio placentae, and fetal growth restriction, and the most common outcome of preeclampsia includes preterm birth, low birth weight, or placental abruption [22]. Perinatal consequences can also include respiratory distress syndrome, admission to the neonatal intensive care unit (NICU), jaundice, and mortality [23,24].

This study aims to retrospectively assess the maternal and fetal outcomes of preeclampsia with and without severe features in King Abdulaziz University Hospital (KAUH), Jeddah. We identified the maternal and fetal outcomes of preeclampsia among pregnant patients in KAUH, evaluated the role of antenatal follow-up on maternal and fetal outcomes of preeclampsia, and recognized the association between the maternal and fetal complications of preeclampsia. We achieved these objectives to prove the significance of antenatal care follow-up.

## Materials and methods

This retrospective study was conducted in KAUH, Jeddah, for three months, and a retrospective chart review was performed for pregnant patients who were admitted to KAUH with the diagnosis of preeclampsia from 2019 to 2021.

We evaluated 83 pregnant women who were diagnosed with preeclampsia from June 2019 to December 2021. We excluded mothers and neonate patients with missing information in medical files. We also analyzed the feto-maternal outcomes for those who were diagnosed with hypertension during pregnancy. Data were collected from the hospital’s electronic database (Phoenix).

The maternal data variables included patients’ demographics, source of admission, gestational age, previous antenatal follow-ups in KAUH, and maternal complications (eclampsia, hypertension, HELLP syndrome, and hydralazine-induced lupus). The neonatal data variables included the neonatal outcomes of preeclampsia (total deceased, intrauterine fetal demise (IUFD), neonatal death, prematurity, intrauterine growth restriction (IUGR), respiratory distress syndrome (RDS), neonatal jaundice, necrotizing enterocolitis, sepsis, hypoglycemia, diarrhea, skin cold to touch, and low birth weight). Preeclampsia was plotted on the chart as preeclampsia with severe features and preeclampsia without severe features, as defined by the International Society for the Study of Hypertension in Pregnancy (ISSHP): “presence of de novo hypertension after 20 weeks gestation accompanied by proteinuria and/or evidence of maternal acute kidney injury (AKI), liver dysfunction, neurological features, hemolysis or thrombocytopenia, or fetal growth restriction.”

Ethical approval was obtained from The Research Ethics Committee, Unit of Biomedical Ethics, King Abdulaziz University, Jeddah. This study was approved by the Institutional Review Board of King Abdulaziz University (reference number: 460-21, September 28, 2021).

## Results

Table 1 presents the descriptive statistics of the gestational age according to preeclampsia with severe features. The mean gestational age for patients with severe features was 33.32 weeks, and the mean gestational age for patients with preeclampsia without severe features was 36 weeks. A t-test verified the difference between the gestational age for women who suffered from preeclampsia with severe features and the gestational age for those who did not suffer from these features at a significant p-value of 0.05.

**Table 1:** Independent samples t-test for gestational age according to preeclampsia with severe features.

	Preeclampsia with severe features	N	Mean	Standard deviation	t-test	
					t-value	Significance
Gestational age	Yes	59	33.32	4.929	2.5	0.018
	No	17	36.00	3.571		

As shown in Table 2 and Table 3, the details demonstrate a crosstabulation with the chi-square test for preeclampsia with/without severe features and the different variables of neonatal outcomes. A highly significant relationship between preeclampsia with severe features and neonatal hypoglycemia (significance = 0.021) was present as it was the least associated neonatal outcome of preeclampsia with severe features. The strength of this relation is a median correlation (phi coefficient = 0.30). No relationships were found between preeclampsia with/without severe features and the other neonatal outcomes, namely, IUFD, neonatal death, prematurity, RDS, etc.

**Table 2:** Cross-tabulation with the chi-square test to see the relationship between preeclampsia with severe features and the different variables of neonatal outcomes. *Significant value at sigma = 0.05.

Variable	Group	Preeclampsia with severe features		Pearson chi-square			Correlation
		Yes	No	Value	df	Significance	
Total deceased	Yes	10 (12.5%)	1 (1.3%)	1.32	1	0.440	-
	No	52 (65.0%)	17 (21.3%)				
Intrauterine fetal demise	Yes	5 (6.3%)	1 (1.3%)	0.13	1	1.000	-
	No	57 (71.3%)	17 (21.3%)				
Neonatal death	Yes	6 (7.5%)	0 (0.0%)	1.88	1	0.328	-
	No	56 (70.0%)	18 (22.5%)				
Prematurity	Yes	16 (20.0%)	6 (7.5%)	0.40	1	0.557	-
	No	46 (57.5%)	12 (15.0%)				
Intrauterine growth restriction	Yes	11 (13.8%)	2 (2.5%)	0.45	1	0.722	-
	No	51 (63.8%)	16 (20.0%)				
Respiratory distress syndrome	Yes	17 (21.3%)	6 (7.5%)	0.24	1	0.626	-
	No	45 (56.3%)	12 (15.0%)				
Neonatal jaundice	Yes	12 (15.0%)	3 (3.8%)	0.07	1	1.000	-
	No	50 (62.5%)	15 (18.8%)				
Necrotizing enterocolitis	Yes	1 (1.3%)	0 (0.0%)	0.29	1	1.000	-
	No	61 (76.3%)	18 (22.5%)				
Sepsis	Yes	2 (2.5%)	0 (0.0%)	0.60	1	1.000	-
	No	60 (75.0%)	18 (22.5%)				
Hypoglycemia	Yes	2 (2.5%)	4 (5.0%)	7.26	1	0.021*	0.30
	No	60 (75.0%)	14 (17.5%)				
Diarrhea	Yes	0 (0.0%)	1 (1.3%)	3.49	1	0.225	-
	No	62 (77.5%)	17 (21.3%)				
Skin cold to touch	Yes	2 (2.5%)	0 (0.0%)	0.60	1	1.000	-
	No	60 (75.0%)	18 (22.5%)				
Low birth weight	Yes	7 (8.8%)	1 (1.3%)	0.51	1	0.676	-
	No	55 (68.8%)	17 (21.3%)				
Others	Yes	15 (18.8%)	4 (5.0%)	0.03	1	1.000	-
	No	47 (58.8%)	14 (17.5%)				

**Table 3:** Cross-tabulation with the chi-square test to see the relationship between preeclampsia without severe features and the different variables of neonatal outcomes. *Significant value at sigma = 0.05.

Variable	Group	Preeclampsia without severe features		Pearson chi-square			Correlation
		Yes	No	Value	df	Significance	
Total deceased	Yes	1 (1.2%)	10 (12.3%)	1.27	1	0.441	-
	No	17 (21.0%)	53 (65.4%)				
Intrauterine fetal demise	Yes	1 (1.2%)	5 (6.2%)	0.12	1	1.000	-
	No	17 (21.0%)	58 (71.6%)				
Neonatal death	Yes	0 (0.0%)	6 (7.4%)	1.85	1	0.329	-
	No	18 (22.2%)	57 (70.4%)				
Prematurity	Yes	6 (7.4%)	16 (19.8%)	0.45	1	0.554	-
	No	12 (14.8%)	47 (58.0%)				
Intrauterine growth restriction	Yes	2 (2.5%)	11 (13.6%)	0.42	1	0.720	-
	No	16 (19.8%)	52 (64.2%)				
Respiratory distress syndrome	Yes	5 (6.2%)	18 (22.2%)	0.01	1	1.000	-
	No	13 (16.0%)	45 (55.6%)				
Neonatal jaundice	Yes	3 (3.7%)	12 (14.8%)	0.05	1	1.000	-
	No	15 (18.5%)	51 (63.0%)				
Necrotizing enterocolitis	Yes	0 (0.0%)	1 (1.2%)	0.29	1	1.000	-
	No	18 (22.2%)	62 (76.5%)				
Sepsis	Yes	1 (1.2%)	2 (2.5%)	0.22	1	0.535	-
	No	17 (21.0%)	61 (75.3%)				
Hypoglycemia	Yes	3 (3.7%)	3 (3.7%)	2.89	1	0.120	-
	No	15 (18.5%)	60 (74.1%)				
Diarrhea	Yes	1 (1.2%)	0 (0.0%)	3.54	1	0.222	-
	No	17 (21.0%)	63 (77.8%)				
Skin cold to touch	Yes	0 (0.0%)	2 (2.5%)	0.59	1	1.000	-
	No	18 (22.2%)	61 (75.3%)				
Low birth weight	Yes	1 (1.2%)	7 (8.6%)	0.49	1	0.677	-
	No	17 (21.0%)	56 (69.1%)				
Others	Yes	3 (3.7%)	16 (19.8%)	0.594	1	0.542	-
	No	15 (18.5%)	47 (58.0%)				

As shown in Table 4, there was no relationship between pregnant patients who followed up antenatally in KAUH and neonatal outcomes (significance = 1.000), and there are no significant correlations between these variables.

**Table 4:** Cross-tabulation with the chi-square test to see the relationship between antenatal follow-ups in King Abdulaziz University Hospital and the different variables of neonatal outcomes.

Variable	Group	Antenatal follow-ups in KAUH		Pearson chi-square			Correlation
		Follow up	Non-follow-up	Value	df	Significance	
Total deceased	Yes	4 (5.0%)	7 (8.8%)	0.25	1	0.726	-
	No	20 (25.0%)	49 (61.3%)				
Intrauterine fetal demise	Yes	3 (3.8%)	3 (3.8%)	1.24	1	0.358	-
	No	21 (26.3%)	53 (66.3%)				
Neonatal death	Yes	1 (1.3%)	5 (6.3%)	0.55	1	0.663	-
	No	23 (28.8%)	51 (63.8%)				
Prematurity	Yes	6 (7.5%)	16 (20.0%)	0.11	1	0.792	-
	No	18 (22.5%)	40 (50.0%)				
Intrauterine growth restriction	Yes	4 (5.0%)	9 (11.3%)	0.01	1	1.000	-
	No	20 (25.0%)	47 (58.8%)				
Respiratory distress syndrome	Yes	5 (6.3%)	17 (21.3%)	0.76	1	0.428	-
	No	19 (23.8%)	39 (48.8%)				
Neonatal jaundice	Yes	4 (5.0%)	11 (13.8%)	0.10	1	1.000	-
	No	20 (25.0%)	45 (56.3%)				
Necrotizing enterocolitis	Yes	0 (0.0%)	1 (1.3%)	0.43	1	1.000	-
	No	24 (30.0%)	55 (68.8%)				
Sepsis	Yes	0 (0.0%)	3 (3.8%)	1.34	1	0.550	-
	No	24 (30.0%)	53 (66.3%)				
Hypoglycemia	Yes	3 (3.8%)	2 (2.5%)	2.29	1	0.156	-
	No	21 (26.3%)	54 (67.5%)				
Diarrhea	Yes	1 (1.3%)	0 (0.0%)	2.36	1	0.300	-
	No	23 (28.8%)	56 (70.0%)				
Skin cold to touch	Yes	1 (1.3%)	1 (1.3%)	0.39	1	0.513	-
	No	23 (28.8%)	55 (68.8%)				
Low birth weight	Yes	0 (0.0%)	8 (10.0%)	3.81	1	0.097	-
	No	24 (30.0%)	48 (60.0%)				
Others	Yes	4 (5.0%)	14 (17.5%)	0.67	1	0.562	-
	No	20 (25.0%)	42 (52.5%)				
Maternal complications	Yes	9 (11.3%)	24 (30.0%)	0.20	1	0.656	-
	No complications	15 (18.8%)	32 (40.0%)				

According to the data in Table 5 and Table 6, there was a highly significant relationship between preeclampsia without severe features and admission source (significance = 0.013), and the strength of this relationship was a median correlation (phi coefficient = 0.31). There was a highly significant relationship between preeclampsia without severe features and antenatal follow-ups in KAUH (significance = 0.001), and the strength of this relationship was a median correlation (phi coefficient = 0.37). There was also a highly significant relationship between preeclampsia without severe features and maternal complications (significance = 0.0001), and the strength of this relationship was a median correlation (phi coefficient = 0.39). No relationships were found for other variables.

**Table 5:** Cross-tabulation with the chi-square test to see the relationship between preeclampsia without severe features, the admission source, the mother’s nationality, antenatal follow-ups, and maternal complications. *Significant value at sigma = 0.05.

Variable	Group	Preeclampsia without severe features		Pearson chi-square			Correlation
		Yes	No	Value	df	Significance	
Admission source	Emergency department	12 (15.0%)	57 (71.3%)	7.51	2	0.013*	0.31
	Non-emergency department	6 (7.5%)	5 (6.3%)				
Mother’s nationality	Saudi	9 (11.3%)	19 (23.8%)	3.46	2	0.177	-
	Non-Saudi	9 (11.3%)	37 (46.3%)				
	Unknown	0 (0.0%)	6 (7.5%)				
Did the patient follow up in KAUH	Follow-up	11 (13.8%)	13 (16.3%)	10.71	1	0.001*	0.37
	Non-follow-up	7 (8.8%)	49 (61.3%)				
Maternal complications	Yes	1 (1.3%)	32 (40.0%)	12.21	1	0.0001*	0.39
	No complications	17 (21.3%)	30 (37.5%)				
Eclampsia	Yes	1 (1.3%)	14 (17.5%)	2.65	1	0.170	-
	No	17 (21.3%)	48 (60.0%)				
Hypertension	Yes	0 (0.0%)	2 (2.5%)	0.60	1	1.000	-
	No	18 (22.5%)	60 (75.0%)				
HELLP syndrome	Yes	0 (0.0%)	5 (6.3%)	1.55	1	0.582	-
	No	18 (22.5%)	57 (71.3%)				
Hydralazine-induced lupus	Yes	0 (0.0%)	1 (1.3%)	0.29	1	1.000	-
	No	18 (22.5%)	61 (76.3%)				
Others	Yes	1 (1.3%)	11 (13.8%)	1.63	1	0.280	-
	No	17 (21.3%)	51 (63.8%)				

**Table 6:** Descriptive analysis for the qualitative variables of maternal nationality.

Variable	Group	Frequency	Percentage (%)
Nationality	Saudi	28	35%
	Non-Saudi	46	57.5%
Different nationalities	Yemeni	9	11.3%
	Myanmar	11	13.8%
	Somalian	7	8.8%
	Ethiopian	4	5%
	Chadian	7	8.8%
	Sudanese	2	2.5%
	Bangladeshi	1	1.3%
	Senegal	2	2.5%
	Indian	1	1.3%
	Nigerian	1	1.3%
	Filipino	1	1.3%
	Unknown	6	7.5%

Table 7 uses the chi-square test to examine whether there is a relationship between preeclampsia with severe features, the admission source, the mother’s nationality, antenatal follow-ups, and maternal complications. There was a highly significant relationship between preeclampsia with severe features and admission source (significance = 0.010) as most cases of preeclampsia with severe features were admitted through the emergency department (72.2%), and the strength of this relationship was a median correlation (phi coefficient = 0.32). There was a highly significant relationship between preeclampsia with severe features and antenatal follow-ups in KAUH (significance = 0.001) as most patients who did not follow up in KAUH developed preeclampsia with severe features (62%), and the strength of this relationship was a median correlation (phi coefficient = 0.39). Moreover, there was a highly significant relationship between preeclampsia with severe features and maternal complications (significance = 0.001) as the percentage of women with preeclampsia with severe features who developed maternal complications (40.5%), while those who did not develop severe features did not have maternal complications, as shown in Table 5. The strength of this relationship was a median correlation (phi coefficient = 0.38).

**Table 7:** Cross-tabulation with the chi-square test to see the relationship between preeclampsia with severe features and the admission source, the mother’s nationality, antenatal follow-ups, and maternal complications. *Significant value at sigma = 0.05.

Variable	Group	Preeclampsia with severe features		Pearson chi-square			Correlation
		Yes	No	Value	df	Significance	
Admission source	Emergency department	57 (72.2%)	11 (13.9%)	8.25	1	0.010*	0.32
	Non-emergency department	5 (6.3%)	6 (7.6%)				
Mother’s nationality	Saudi	19 (24.1%)	8 (10.1%)	2.80	2	0.246	-
	Non-Saudi	37 (46.8%)	9 (11.4%)				
	Unknown	6 (7.6%)	0 (0.0%)				
Did the patient follow up in KAUH	Follow-up	13 (16.5%)	11 (13.9%)	12.07	1	0.001*	0.39
	Non-follow-up	49 (62.0%)	6 (7.6%)				
Maternal complications	Yes	32 (40.5%)	1 (1.3%)	11.47	1	0.001*	0.38
	No Complications	30 (38.0%)	16 (20.3%)				
Eclampsia	Yes	14 (17.7%)	1 (1.3%)	2.42	1	0.170	-
	No	48 (60.8%)	16 (20.3%)				
Hypertension	Yes	2 (2.5%)	0 (0.0%)	0.56	1	1.000	-
	No	60 (75.9%)	17 (21.5%)				
HELLP syndrome	Yes	5 (6.3%)	0 (0.0%)	1.46	1	0.579	-
	No	57 (72.2%)	17 (21.5%)				
Hydralazine-induced lupus	Yes	1 (1.3%)	0 (0.0%)	0.28	1	1.000	-
	No	61 (77.2%)	17 (21.5%)				
Others	Yes	11 (13.9%)	1 (1.3%)	1.46	1	0.445	-
	No	51 (64.6%)	16 (20.3%)				

As shown in Table 8, there was no relationship between the admission source and the mother’s nationality (significance = 0.263), and there was no significant correlation between these two variables. There was no relationship between admission source and maternal complications (significance = 0.113), and there was no significant correlation between these two variables. A highly significant relationship between admission source and antenatal follow-ups in KAUH (significance = 0.0001) was found, as the percentage of women who did not follow up antenatally in KAUH and were admitted through the emergency department (70%), and the strength of this relationship was a median correlation (phi coefficient = 0.61).

**Table 8:** Cross-tabulation with the chi-square test to see the relationship between admission source and maternal nationality, maternal complications, and antenatal follow-up in King Abdulaziz University Hospital. *Significant value at sigma = 0.05.

Variables	Group	Admission source		Pearson chi-square			Correlation
		Emergency department	Non-emergency department	Value	df	Significance	
Mother’s nationality	Saudi	22 (27.5%)	6 (7.5%)	2.67	2	0.263	-
	Non-Saudi	41 (51.3%)	5 (6.3%)				
	Unknown	6 (7.5%)	0 (0.0%)				
Maternal complications	Yes	31 (38.8%)	2 (2.5%)	2.80	1	0.113	-
	No complications	38 (47.5%)	9 (11.3%)				
Did the patient follow up in KAUH	Follow-up	13 (16.3%)	11 (13.8%)	29.76	1	0.0001*	0.61
	Non-follow-up	56 (70.0%)	0 (0.0%)				

As shown in Table 9, there was a significant relationship between antenatal follow-ups in KAUH and the mother’s nationality (significance = 0.030), and the strength of this relationship was a median correlation (Cramer’s V coefficient = 0.30) as 43.8% of the patients who did not follow up in KAHU were non-Saudis.

**Table 9:** Cross-tabulation with the chi-square test to see the relationship between antenatal follow-ups in King Abdulaziz University Hospital and the mother’s nationality. *Significant value at sigma = 0.05.

Variable	Group	Did the patient follow up in KAUH		Pearson chi-square			Correlation
		Follow-up	Non-follow-up	Value	df	Significance	
Mother’s nationality	Saudi	13 (16.3%)	15 (18.8%)	6.98	2	0.030*	0.30
	Non-Saudi	11 (13.8%)	35 (43.8%)				
	Unknown	0 (0.0%)	6 (7.5%)				

## Discussion

In this study, we discussed the neonatal outcomes of preeclampsia (total deceased, IUFD, neonatal death, prematurity, IUGR, RDS, neonatal jaundice, necrotizing enterocolitis, sepsis, hypoglycemia, diarrhea, skin cold to touch, and low birth weight) in association with preeclampsia with and without severe features. There was a significant relationship between preeclampsia with severe features and neonatal hypoglycemia as the data showed neonatal hypoglycemia is the least associated neonatal outcome of preeclampsia with severe features. This differs from a previous study [25] which reported that the risk of postpartum neonatal hypoglycemia was significantly higher among the preeclampsia cases (p = 0.001). Conversely, another study did not investigate postpartum neonatal hypoglycemia as one of the neonatal outcomes of preeclampsia with and without severe features [22].

In the studied maternal complications (eclampsia, hypertension, HELLP syndrome, and hydralazine-induced lupus), we could not find an association between preeclampsia with severe features and a specific maternal complication. However, preeclampsia with severe features is associated with overall maternal complications by 40.5%. While those who did not develop severe features did not develop maternal complications (1.3%). This agrees with the study [26] which reported that women with persistent hypertension can be provided appropriate admission into care and permit possible treatment that may aid in averting some of the long-term cardiovascular morbidity that is seen among women with preeclampsia.

In this study, 62% of the patients who did not visit for antenatal follow-up during their pregnancies developed preeclampsia with severe symptoms, 43.8% of the patients who did not follow up antenatally were non-Saudi, and 72% of the patients who are diagnosed with preeclampsia with severe features and did not have previous antenatal follow up visits were evaluated and admitted through emergency department. This is consistent with the fact that the patients who did not follow up antenatally did not have the opportunity to book for delivery, presenting to the emergency department with severe features. Thus, who do not follow up antenatally are more likely to develop preeclampsia with severe features, which agrees with the previous study [27], where patients with postpartum preeclampsia/eclampsia did not have a history of preeclampsia during pregnancy. Therefore, close postpartum follow-up is necessary to successfully manage and perhaps reduce a woman’s long-term risk.

Not all women with this diagnosis who report to the emergency department in the postpartum period exhibit all classic symptoms of this disease, such as hypertension, edema, proteinuria, and hyperreflexia. A comparative study reported that management, following up, and referral of pregnant women are critical to reducing both mortality and morbidity rates in both early and late-onset preeclampsia [28]. Similarly, a subsequent study [29] utilized a different database and a larger sample size of systemic lupus erythematosus patients who had a threefold greater risk of preeclampsia, a 20-fold increased risk of maternal mortality, and an increased incidence of infections and thrombosis.

## Conclusions

The morbidity of preeclampsia with severe features depends on maternal preeclampsia complications and is less associated with unfavorable fetal outcomes. The studied fetal complications were not associated with following up. Although continuous surveillance does not have a direct effect on fetal outcomes, it improves maternal outcomes. Therefore, women need to have a better understanding of the warning signs, and patients at risk of preeclampsia should be counseled. Healthcare providers need to recognize the significance of early screening and taking a patient’s medical history, measuring blood pressure, and providing consistent and effective management according to international and national guidelines to reduce the likelihood of unfavorable outcomes. As for unspecialized healthcare centers, it is imperative to identify symptoms early on and refer patients in a timely manner. For further research, we suggest ranking maternal outcomes by the most common presentations to the emergency department by increasing the sample size and applying the study to multiple centers. Furthermore, correlating the maternal outcomes of preeclampsia with severe features with maternal age might help in emphasizing the importance of antenatal follow-up for specific pregnant age groups. Additionally, conducting cross-sectional studies in different regions to determine the awareness level of maternal and fetal preeclampsia outcomes will help in establishing proper preventive measures.
